# Supplementary material for: Developmental and Neurotoxicity of Acrylamide to Zebrafish
Source: Int J Mol Sci. 2021 Mar 29;22(7):3518. doi: 10.3390/ijms22073518 (PMC8037265; doi:10.3390/ijms22073518)

Developmental and neurotoxicity of acrylamide to zebrafish

Jong-Su Park[1,†], Palas Samanta[1,2,†], Sangwoo Lee[1], Jieon Lee[1], Jae-Woo Cho[3], Hang-Suk Chun[1], Seokjoo Yoon[4], Woo-Keun Kim[1,*]

[1]Biosystem Research Group, Korea Institute of Toxicology, Daejeon 34111, South Korea;

jongsu.park@pitt.edu (J.-S.P.); sangwoo.lee@kitox.re.kr (S.W.L.);

jieon.lee@kitox.re.kr (J.E.L.); hangsuk.chun@kitox.re.kr (H.-S.C.)

[2]Department of Environmental Science, Sukanta Mahavidyalaya, University of North Bengal, Dhupguri, West Bengal, India;

samanta.palas2010@gmail.com (P.S.)

[3]Toxic Pathology Research Group, Korea Institute of Toxicology, Daejeon 34111, South Korea;

cjwoo@kitox.re.kr (J.-W.C.)

[4]Molecular Toxicity Research Group, Korea Institute of Toxicology, Daejeon 34111, South Korea;

sjyoon@kitox.re.kr (S.J.Y.)

[†]These authors contributed equally to the work.

* Corresponding author: wookkim@kitox.re.kr (W.-K.K.)

Figure S1. Comparison of developmental morphology. Acrylamide treated zebrafish larvae showed swim bladder deficiency (white arrow), heart edema (black arrowhead).

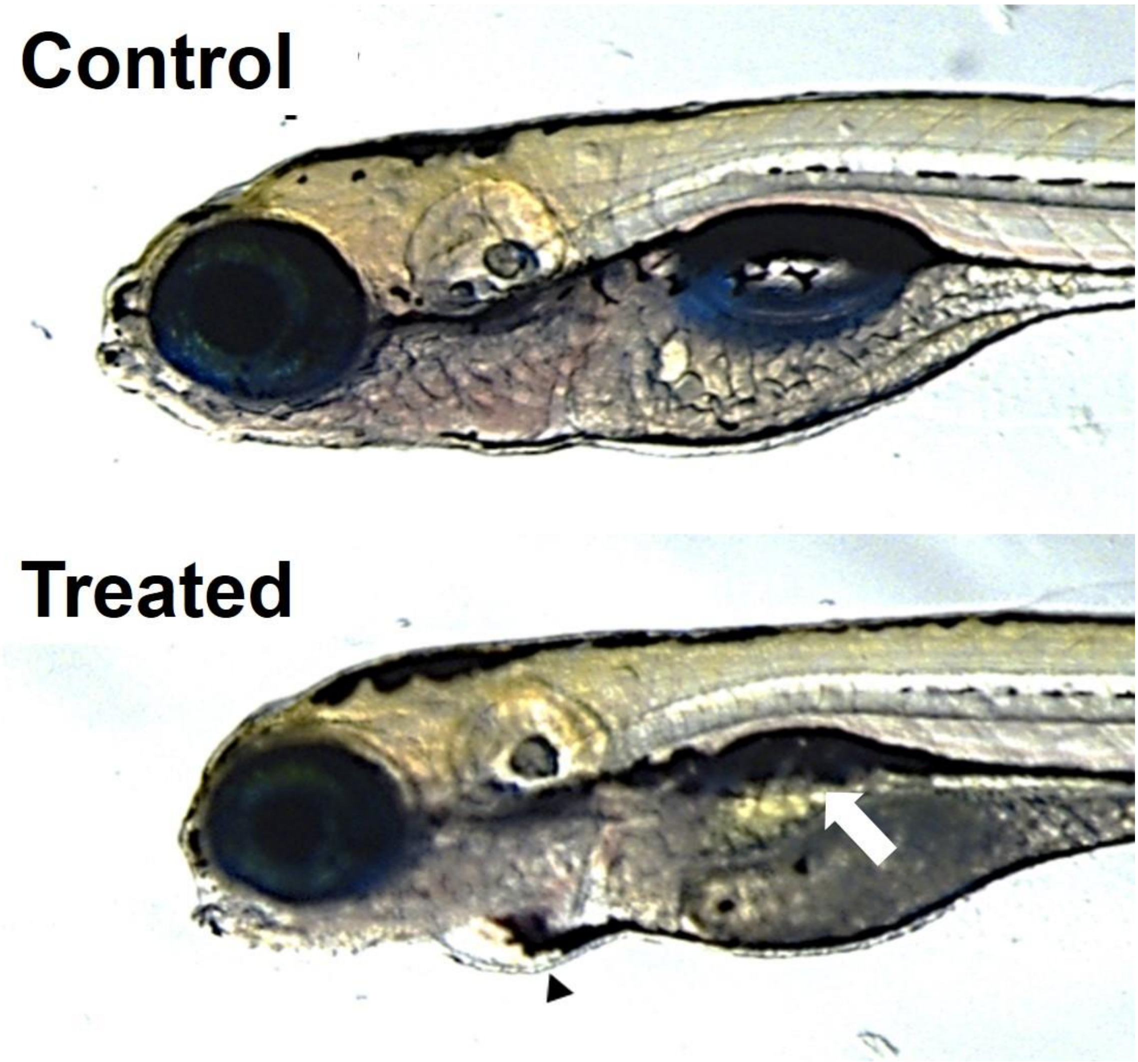

Figure S2. Myelinating, mature oligodendrocytes and Schwann cells showed normal development under acrylamide treatment. To investigate whether acrylamide treatment can affect myelination-related neuron impairment, we used *tg*(*mbp:mGFP*), which expressed fluorescence in myelinating, mature oligodendrocytes and Schwann cells of zebrafish. However, no differences from controls were detected in acrylamide treated groups.

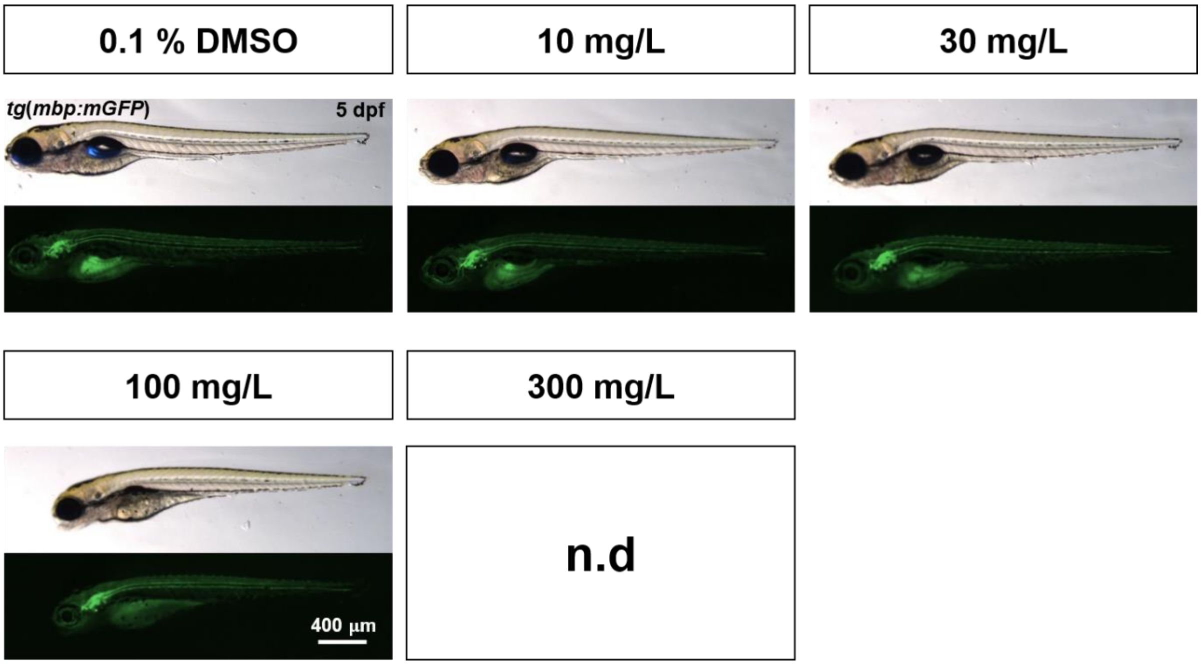

Figure S3. The anterior lateral line showed normal differentiation. The cranial anterior lateral line showed no differences between 0.1 % DMSO and acrylamide treated groups (white arrow).

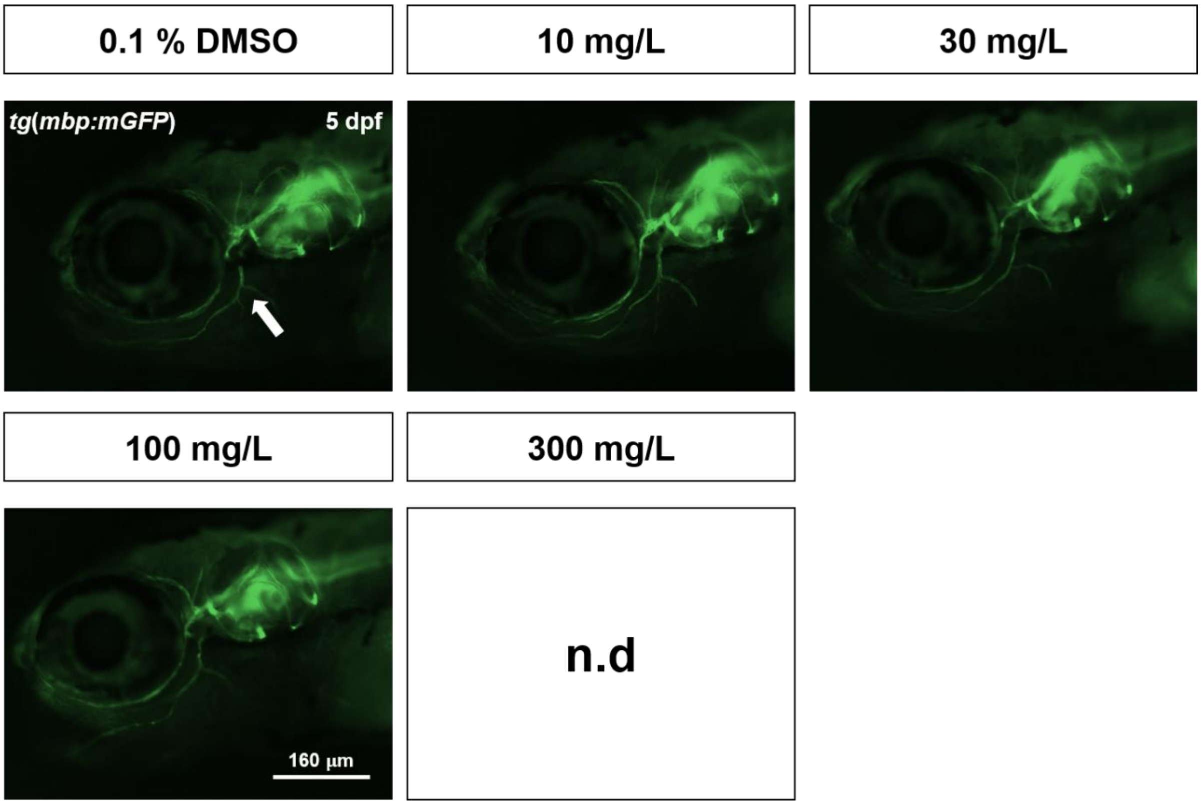

Figure S4. Neural crest origin cells showed normal development. To investigate whether acrylamide can affect neural crest origin cells, we used *tg*(*sox10:eGFP*), which expresses fluorescence in neural crest origin cells. However, no differences were detected in acrylamide treated groups compare with the controls.

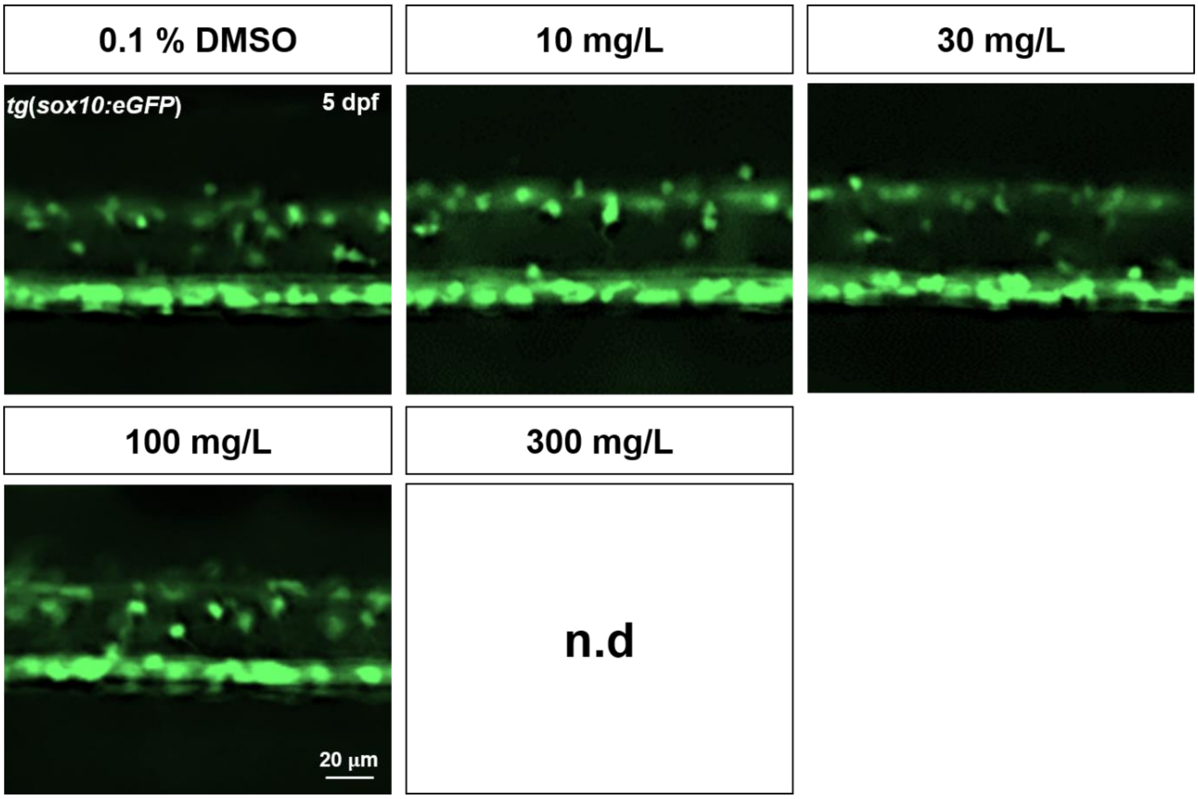

Supplement: Supplementary file 1 [file ijms-22-03518-s001.zip › ijms-1131742/Supplementary material_IJMS.pdf]
